# Effectiveness and success factors of educational inhaler technique interventions in asthma & COPD patients: a systematic review

**DOI:** 10.1038/s41533-017-0022-1

**Published:** 2017-04-13

**Authors:** Sven L. Klijn, Mickaël Hiligsmann, Silvia M. A. A. Evers, Miguel Román-Rodríguez, Thys van der Molen, Job F. M. van Boven

**Affiliations:** 1 Department of Health Services Research, CAPHRI, Maastricht University, P.O. Box 616, 6200 Maastricht, The Netherlands; 2 Son Pisa Primary Health Care Centre, Balearic Health Service, Palma de Mallorca, Spain; 3 Department of General Practice, Groningen Research Institute for Asthma and COPD (GRIAC), University Medical Centre Groningen, University of Groningen, Groningen, The Netherlands

## Abstract

With the current wealth of new inhalers available and insurance policy driven inhaler switching, the need for insights in optimal education on inhaler use is more evident than ever. We aimed to systematically review educational inhalation technique interventions, to assess their overall effectiveness, and identify main drivers of success. Medline, Embase and CINAHL databases were searched for randomised controlled trials on educational inhalation technique interventions. Inclusion eligibility, quality appraisal (Cochrane’s risk of bias tool) and data extraction were performed by two independent reviewers. Regression analyses were performed to identify characteristics contributing to inhaler technique improvement. Thirty-seven of the 39 interventions included (95%) indicated statistically significant improvement of inhaler technique. However, average follow-up time was relatively short (5 months), 28% lacked clinical relevant endpoints and all lacked cost-effectiveness estimates. Poor initial technique, number of inhalation procedure steps, setting (outpatient clinics performing best), and time elapsed since intervention (all, *p* < 0.05), were shown to have an impact on effectiveness of the intervention, explaining up to 91% of the effectiveness variation. Other factors, such as disease (asthma vs. chronic obstructive pulmonary disease), education group size (individual vs. group training) and inhaler type (dry powder inhalers vs. pressurised metered dose inhalers) did not play a significant role. Notably, there was a trend (*p* = 0.06) towards interventions in adults being more effective than those in children and the intervention effect seemed to wane over time. In conclusion, educational interventions to improve inhaler technique are effective on the short-term. Periodical intervention reinforcement and longer follow-up studies, including clinical relevant endpoints and cost-effectiveness, are recommended.

## Introduction

Bronchodilators and corticosteroids play a key role in maintaining disease control in asthma and chronic obstructive pulmonary disease (COPD) patients. Delivery of these drugs is mainly achieved by inhalers, which can be categorised into three types: pressurised metered dose inhalers (pMDIs), dry powder inhalers (DPIs) and nebulisers. Previous studies, performed in controlled settings, showed that all inhalers are equally capable of delivering an appropriate medication dose.^1, 2^ In daily use however, a large majority of patients make inhalation errors.^3^ Suboptimal inhaler technique is associated with worsened health outcomes, such as increased risk of hospitalisation and poor disease control.^4–6^ Consequences can also be found in the financial context as studies estimate that a considerable amount of resources spent on inhalers are wasted.^7^ Important inter-patient differences have repeatedly been shown, with as few as 25% of the patients able to demonstrate a correct technique.^8–11^ As such, it is of utmost importance to properly train patients on inhaler technique.^4, 12, 13^ Various educational interventions to do so have been reported. However, so far there has been no systematic review of these interventions, leaving the key characteristics of successful interventions to remain obscure. With little improvement shown over time,^14^ the current wealth of new inhalers available and frequent health insurance policy driven inhaler switches, the need for optimal education on inhaler use is more evident than ever.

This review aims to provide a systematic overview of educational interventions focusing on inhaler technique in asthma and COPD patients, assess their overall effectiveness, and identify their main drivers of success.

## Results

### Inclusion

**Fig. 1:**
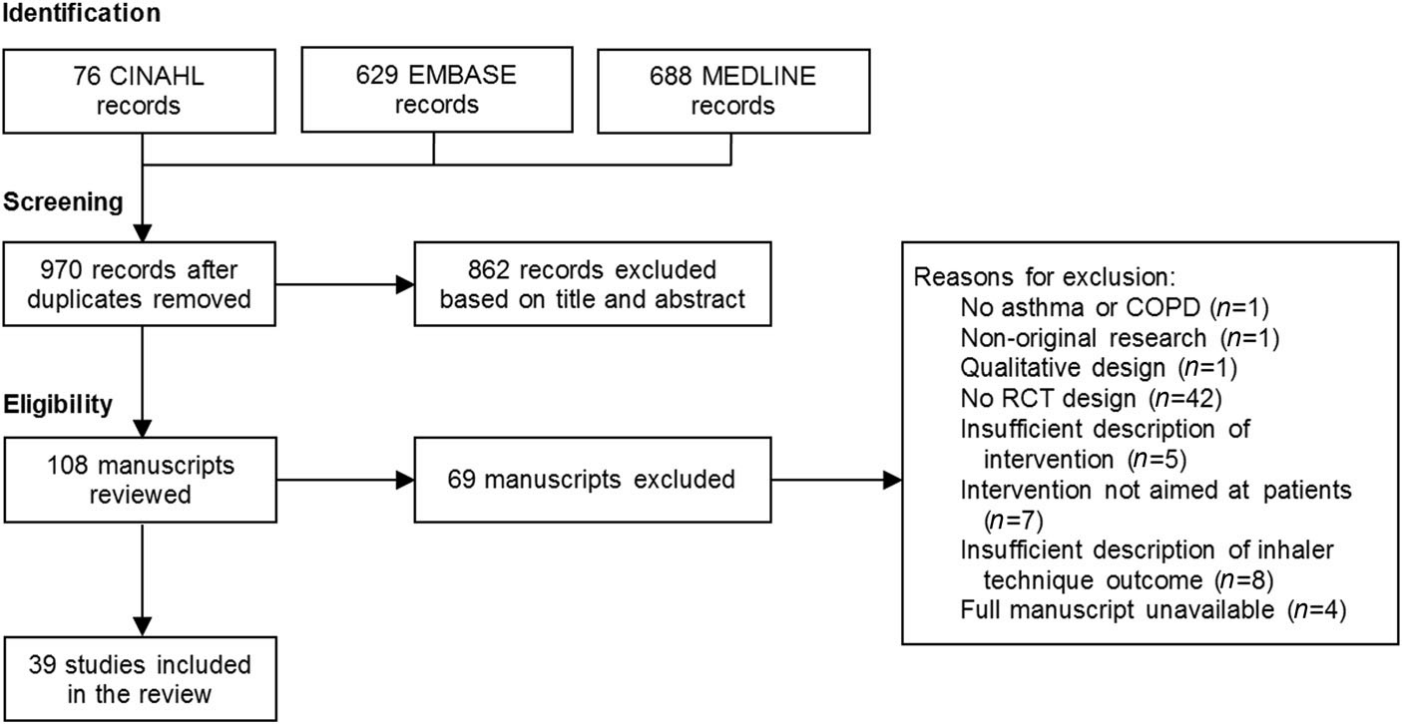
Flow diagram on article inclusion

The literature search yielded a total of 1393 results. Of the 970 unique articles, 862 were excluded based on title and abstract, while a further 69 articles were excluded during full-text screening (Fig. 1). Initial agreement between reviewers on eligibility was 87% (Cohen’s κ = 0.72). After one consensus round, full agreement was reached (e-Appendix 2) and 39 articles were eventually included.^15–53^ Full manuscripts of four studies were unavailable, all dating from 2001 or before.^54–57^ Three authors were contacted, but did not reply. The remaining author could not be traced.

### Study quality

**Fig. 2:**
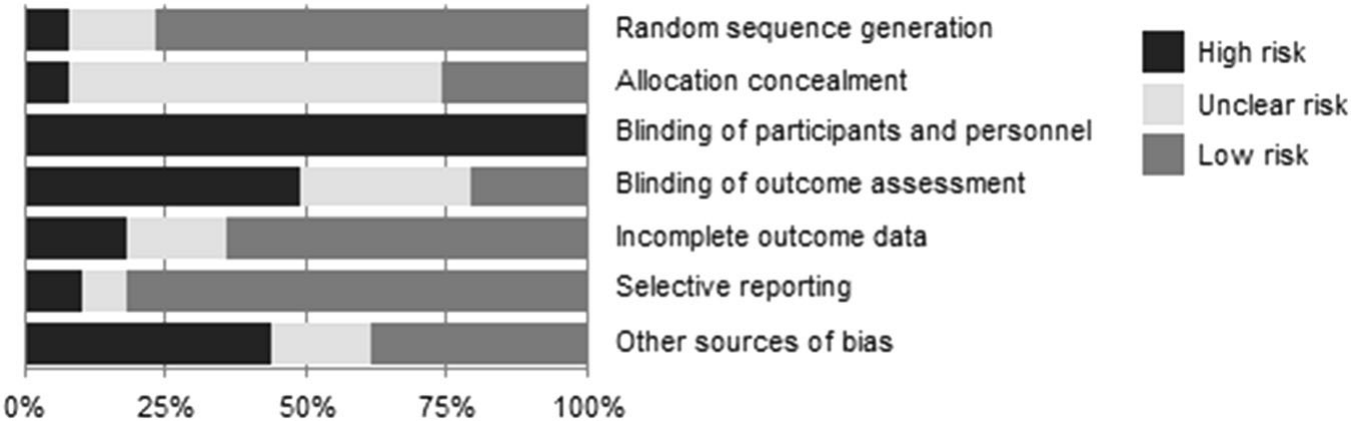
Quality assessment of included studies. *Percentages* represent the percentage of included articles having a high risk (*black bar*), unclear risk (*light grey bar*) or low risk (*medium-grey bar*) of bias for each category in the Cochrane Collaboration’s risk of bias assessment tool

Inter-reviewer agreement on the study quality assessment was of moderate strength (Cohen’s weighted κ = 0.51), but consensus was reached after one consensus round. Twelve of the 39 studies scored a low risk of bias on four or more of the seven categories (see e-Appendix 3). Random sequence generation and selective reporting were found to be best addressed by the studies (Fig. 2). Allocation concealment was frequently not described, and it was therefore difficult to determine whether concealment was sufficient but not reported, or insufficient and a potential source of bias. As was already established beforehand, blinding of participants was not possible, which is reflected in the quality appraisal results. Blinding of outcome assessment was possible, but in almost half of the studies not implemented. Regression analysis showed that quality of the study was not associated with intervention outcome results (*p* > 0.05), irrespective of the type of outcome reported.

### Study characteristics

An overview of the 39 studies and their 56 intervention groups is provided in Table 1. Full details of the studies can be found in e-Appendix 4. The majority covered patients with asthma (*n* = 35), of which six also included COPD patients. Studies exclusively performed in COPD-patients were rare (*n* = 4). The interventions mainly took place in outpatient clinics (*n* = 17) or community pharmacy settings (*n* = 15).

**Table 1:** Study and intervention characteristics

FFirst author	Disease	Setting	Sessions	Session length (h:mm)	Delivery	Deliverer	N	Inhaler type	Maximum follow-up (months)	Outcome type	Inhaler technique Improvement		Clinical outcomes
											(%)	(p.p.)	
Al-Showair 2007^15^	Asthma	Outpatient clinic	1	–	Individual	–	107	MDI	1.5	IFR	–		Peak flow:↑, AQLQ: ↑
Armour 2013^16^	Asthma	Pharmacy	4	00:26	Individual	Pharmacist	398	MDI	6	Patients		50 p.p.	ACQ:≈
Basheti 2008^17^	Asthma	Pharmacy	4	00:03	Individual	Pharmacist	97	DPI	6	Steps	49%		Asthma severity: ↓
Basheti 2005^18^	Asthma	Pharmacy	1	00:08	Individual	Researcher	17	DPI	0.5	Steps	75%		None
	Asthma	Pharmacy	1	00:08	Individual	Researcher	17	DPI	0.5	Steps	80%		
Bosnic-Anticevich 2010^19^	Both	Pharmacy	3	–	Individual	Researcher	52	MDI	4	Steps	38%		None
Bynum 2001^20^	Asthma*	Outpatient clinic	1	00:15	Individual	Pharmacist	49	MDI	1	Steps	77%		None
Carpenter 2015^21^	Asthma*	Outpatient clinic	1	00:03	Individual	Researcher	91	MDI	1	Steps	16%		ACT: ≈
Chan 2007^22^	Asthma*	Outpatient clinic	5	–	Individual	Other	60	MDI	12	Steps	8%		QOL: ≈, hospitalisations: ≈
	Asthma*	Outpatient clinic	5	–	Individual	Other	60	DPI	12	Steps	12%		
Chan 2003^23^	Asthma*	Outpatient clinic	4	–	Individual	Other	10	Both	6	Steps	–		Peak flow: ↑, QOL: ≈
Cicutto 2013^24^	Asthma*	School	6	00:53	Group	–	1316	–	12	Steps	48%		QOL:↑, Urgent care:↓
Cordina 2001^25^	Asthma	Pharmacy	1	–	Individual	–	152	–	12	Patients		36 p.p.	QOL: ↑, peak flow: ↑, hospitalisations: ↓
Crane 2014^26^	Asthma	–	1	–	Individual	–	123	Both	12	Patients		16 p.p.	None
De Blaquiere 1989^27^	Both	Outpatient clinic	1	00:17	Individual	Researcher	100	MDI	2	Patients		81 p.p.	Hospitalisations: ≈
De Oliveira 1999^28^	Asthma	Outpatient clinic	8	–	–	Researcher	42	MDI	6	Patients		73 p.p.	ER visits:↓, symptoms:↓, QOL:↑
Garcia-Cardenas 2013^29^	Asthma	Pharmacy	3	–	Individual	Pharmacist	336	DPI	6	Patients		56 p.p.	ACQ:↑
Goodyer 2006^30^	Asthma	–	1	–	Individual	Pharmacist	35	MDI	0	Steps	–		None
	Asthma	–	1	–	Individual	Pharmacist	34	MDI	0	Steps	–		
Goris 2013^31^	COPD	Outpatient clinic	1	–	Individual	–	24	MDI	3	Steps	100%		QOL:↑, attacks:↓, hospitalisations:≈
	COPD	Outpatient clinic	1	–	Individual	–	110	DPI	3	Steps	43%		
Hesselink 2004^32^	Both	Other	2	00:30	Individual	Nurse	276	–	24	Patients		–	QOL: ≈
Horner 2008^33^	Asthma*	School	16	00:15	Group	Other	183	MDI	1.5	Steps	36%		None
Kiser 2012^34^	COPD	Hospital	1	00:23	Individual	Researcher	99	MDI	1.25	Steps	29%		None
	COPD	Hospital	1	00:23	Individual	Researcher	41	DPI	1.25	Steps	22%		
	COPD	Hospital	1	00:23	Individual	Researcher	27	DPI	1.25	Steps	20%		
Kools 2006^35^	Asthma	–	1	00:01	Individual	Researcher	50	MDI	0	Steps	–		None
Kritikos 2007^36^	Asthma	Pharmacy	1	02:30	Group	Pharmacist	22	MDI	3	Patients		73 p.p.	Poor control: ↓, AQOL: ↑
	Asthma	Pharmacy	1	02:30	Group	Pharmacist	25	DPI	3	Patients		79 p.p.	
	Asthma	Pharmacy	1	02:30	Group	Researcher	20	MDI	3	Patients		86 p.p.	
	Asthma	Pharmacy	1	02:30	Group	Researcher	26	DPI	3	Patients		84 p.p.	
Kumar 2009^37^	Both	Hospital	4	–	Individual	Pharmacist	98	MDI	2	Steps	115%		FEV_1_:↑
	Both	Hospital	4	–	Individual	Pharmacist	18	DPI	2	Steps	100%		
Martin 2015^38^	Asthma*	Other	4	–	Individual	Nurse	51	–	12	Steps	50%		Control: ≈
	Asthma*	Other	4	–	Individual	Nurse	50	–	12	Steps	29%		
Mehuys 2008^39^	Asthma	Pharmacy	3	–	Individual	Pharmacist	201	Both	6	Steps	25%		Nighttime symptoms:↓, ACT:↑
Mulloy 1996^40^	Asthma	Outpatient clinic	1	–	Individual	Nurse	60	–	12	Steps	20%		Symptoms: ↓, peak flow: ≈
Patterson 2005^41^	Asthma*	School	8	–	Group	Nurse	173	–	4	Patients		38 p.p.	QOL: ≈
Perneger 2002^42^	Asthma	Hospital	3	01:15	Group	Other	131	–	6	Patients		28 p.p.	QOL: ≈, healthcare utilisation: ≈
Petkova 2008^43^	Asthma	Pharmacy	5	–	–	Researcher	50	–	4	Patients		14 p.p.	Hospitalisation: ↓, QOL:↑
Press 2012^44^	Both	Hospital	1	00:06	Individual	Researcher	50	MDI	0	Patients		52 p.p.	Health-related events:↓
	Both	Hospital	1	00:06	Individual	Researcher	18	DPI	0	Patients		50 p.p.	
Rahmati 2014^45^	Asthma	–	3	–	Group	–	60	MDI	1	Steps	60%		Peak flow: ↑
	Asthma	–	3	–	Group	–	60	MDI	1	Steps	90%		
Rootmensen 2008^46^	Both	Outpatient clinic	1	00:45	Individual	Nurse	191	–	6	Steps	3%		QOL: ≈, Exacerbations: ↓
Rydman 1999^47^	Asthma	Outpatient clinic	1	–	Individual	Researcher	68	MDI	3	Patients		36 p.p.	None
Santos 2010^48^	Asthma	Outpatient clinic	2	01:00	Individual	Pharmacist	28	MDI	2	Steps	167%		None
	Asthma	Outpatient clinic	2	01:00	Individual	Pharmacist	28	DPI	2	Steps	33%		
Tommelein 2014^49^	COPD	Pharmacy	2	00:20	Individual	Pharmacist	734	Both	3	Steps	38%		Hospitalisations↓
Toumas-Shehata 2014^50^	Asthma	Pharmacy	1	–	Individual	Pharmacist	101	DPI	1	Steps	40%		ACQ:↑
Van der Palen 1997^51^	COPD	Outpatient clinic	1	00:45	Group	Nurse	70	Both	9	Steps	26%		None
	COPD	Outpatient clinic	1	00:45	Individual	Other	73	Both	9	Steps	18%		
	COPD	Other	1	00:45	Individual	Other	71	Both	9	Steps	26%		
Verver 1996^52^	Asthma	Other	1	–	Individual	Nurse	48	DPI	0.5	Steps	9%		Dyspnoea: ↓
Wilson 1993^53^	Asthma	Hospital	4	00:45	Individual	Nurse	227	MDI	12	Steps	–		Asthma status: ↑, physical activity: ↑, Acute visits:↓
	Asthma	Hospital	4	01:30	Group	Nurse	229	MDI	12	Steps	–		

*: children, ↑: increase/improvement, ↓: decrease/worsening, ≈: no difference, Outcome type: mean number or percentage of correct steps (in the table: “Steps”), the percentage of patients who showed a correct technique (in the table: “Patients”), or inhalation flow rate (IFR). Improvement over baseline is either reported as percentage (%) or as percentage points (p.p.)

Sample sizes ranged from 10 to 1316 participants with a median of 60 participants. One fifth of the studies targeted children. Of the studies that reported the included inhaler types, 82.8% included pMDIs, whereas DPIs were included in 58.6% of the studies. Ten studies did not specify which inhaler types were included. The average follow-up time was five months; six studies had ≥1 year follow-up.

Outcomes were most frequently recorded as *correct-steps* (64.1%), whereas *correct-patients* outcome reporting was less common (33.3%). One study reported outcomes as improvements in inhalation flow rate.^15^ Improvements over baseline displayed a large difference between studies, with *correct-patients* studies reporting improvements of 3% to 167% and *correct-steps* studies 14 to 86 percentage points. Eleven studies (28%) did not report any relevant clinical outcomes besides inhalation scores.

### Educational interventions

Almost all interventions (89%) included a physical or video demonstration of inhaler use. Physical demonstrations were most common, whereas video demonstrations were used in six studies.^21, 25, 30, 31, 40, 51^ The form of the demonstration did not have a significant effect on improvement of inhaler technique over baseline (*p* > 0.05). Whether or not patients were requested to demonstrate own inhaler use after demonstration was frequently not reported.

Approximately half of the studies (*n* = 22) provided additional disease education or embedded the inhaler education in a more complex intervention. Disease education usually addressed topics such as disease pathophysiology^49^ and disease triggers.^16^ Complex interventions also included counselling on self-management skills^38^ and health beliefs.^29^

The mean number of sessions was 2.6. The mean duration of a session was 30 min, excluding an outlier.^36^ The interventions in outpatient clinics and pharmacy settings were similar in the sense that they were mostly individual educational interventions. Furthermore, the mean number of sessions (outpatient clinics:2.6; pharmacies:2.7) and total intervention time (both 1.5 h) did not statistically differ (*p* > 0.05). However, videos and internet-based education were more common in outpatient settings.^20–23, 31, 40^

### Inhaler technique improvement

Over 90% of studies reported a significant improvement in inhaler technique after intervention. Two studies reported no effect over usual care.^27, 46^ These studies were both single-session interventions in outpatient clinics. Martin et al.^38^ reported a significant improvement only in a subgroup. Younger children did not have significant changes, whereas inhaler technique in older children significantly improved. Several studies reported a (partial) loss of effect of the intervention over time.^17, 19, 24, 47^ This waning effect did not seem to be related to the intervention’s characteristics, the setting in which it was performed, or any patient characteristics. The study with the longest follow-up time showed in a subgroup analysis that patients who attended multiple sessions had an increased inhaler technique over patients who only attended one session.^32^

Regression models (Table 2) showed that several intervention characteristics influenced the intervention’s effectiveness. For *correct-step* studies (*n* = 21), using a forward selection procedure, these were the total number of steps evaluated, setting (outpatient clinics performing best, community pharmacies and non-categorised settings performing worst), adults improved more than children, and baseline performance. The model had an excellent fit (adjusted *R* ^2^: 0.906). Using a backward selection procedure, the total number of steps evaluated and the baseline performance were the only study characteristics that showed a significant influence on the intervention’s effectiveness. For *correct-patients* studies (*n* = 12, with 16 intervention groups), the percentage of patients with baseline correct technique, and follow-up time were significant. Both selection procedures, forward selection and backward elimination, led to the same result and the model had a good fit (adjusted *R* ^2^: 0.862). In both models, publication year, general disease education, number of sessions, session length, total length of intervention, delivery form, sample size, disease, inhaler and gender did not significantly influence improvement inhaler technique improvement.

**Table 2:** Linear regression models with improvement over baseline as dependent variable

	*Correct-steps* interventions (*n* = 32)				*Correct-patients* interventions (*n* = 16)			
	β	95% CI		*p*-value	β	95% CI		*p*-value
		min	max			min	max	
Total number of steps evaluated	0.065	0.027	0.104	0.002				
Intervention setting								
Community pharmacy	[ref]							
Hospital	0.089	−0.051	0.228	0.200				
Outpatient clinic	0.149	0.024	0.274	0.022				
School	0.025	−0.224	0.275	0.835				
Other	−0.004	−0.172	0.164	0.958				
Age group (adults vs. children)	0.153	−0.003	0.310	0.055				
Baseline performance	−2.720	−3.101	−2.338	<0.001	−1.498	−1.921	−1.075	<0.001
Intervention provider								
Pharmacist					[ref]			
Researcher					−0.052	−0.190	0.087	0.414
Nurse					−0.224	−0.450	0.001	0.051
Other					−0.226	−0.458	0.007	0.056
Follow-up time					−0.034	−0.068	−0.001	0.046

**Fig. 3:**
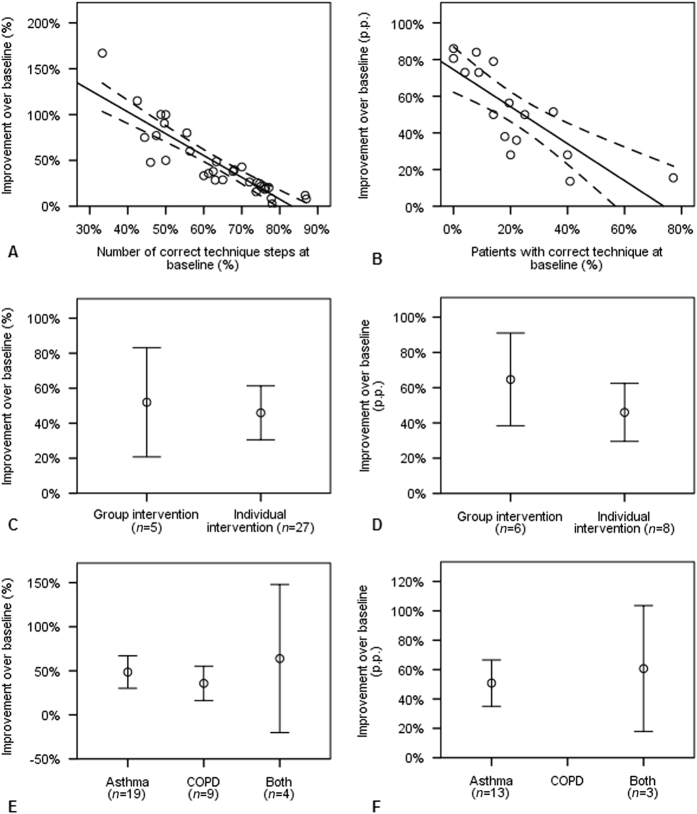
Improvement in inhaler technique plotted against baseline performance (**a**, **b**), type of intervention (**c**, **d**), and patients’ disease background (**e**, **f**) with 95% confidence intervals. The left column (**a**, **c**, and **e**) displays results for *correct-steps* studies, the right column shows results for *correct-patients* studies

Baseline performance explained a large percentage of intervention’s effectiveness, independent of outcome measurement used (Fig. 3a, b). Patients with good baseline technique showed little improvement after intervention. Delivery form (group or individual) was not significantly correlated to inhaler technique improvement (Fig. 3c, d). However, only few interventions were delivered to a group (*n* = 11), whereas the majority was delivered to individuals (*n* = 35). Differences between diseases were difficult to determine, as there were no *correct-patients* studies in COPD-patients and confidence intervals were large (Fig. 3e, f).

### Clinical outcomes

Twenty-eight studies (72%) reported additional clinical outcomes (Table 1). Those outcomes included a measure of control or quality of life (44%), lung function (FEV_1_, peak flow) (15%), symptoms (e.g. night-time symptoms/dyspnoea) (10%), healthcare utilisation (e.g. ER visits, hospitalisations) (28%). Cost-effectiveness was never reported. The majority indicated favourable results for the intervention group, with highest discrepancy regarding effects on quality of life.

## Discussion

### Main findings

This review showed that educational interventions on inhaler technique are effective, at least on the short term. All studies showed improvements and 95% indicated statistical significance with a mean intervention time of 30 min and an average follow-up of five months. Regression analysis revealed several key characteristics that influenced intervention’s effectiveness. Major predictors for success were low baseline performance, outpatient setting and short follow-up time, with setting only being significant when outcomes were assessed in terms of correct number of inhalation steps. Other factors that predicted effectiveness were higher number of steps evaluated, and higher age group. Duration of the intervention, scale (group or individual), executor (pharmacist, nurse or other), inhaler (pMDI or DPI) and disease (asthma or COPD) were not associated with intervention effectiveness. Of note, a trend (*p* = 0.06) was observed in interventions being more effective in adults than in children, however relatively few studies targeted children specifically. The studies that included clinical relevant endpoints mostly indicated favourable clinical effects with highest discrepancy regarding effects on quality of life. Cost-effectiveness was never reported.

### Interpretation of findings in relation to previously published work

There are several factors which were found to explain the variation in improvement of inhaler technique. Of these factors, baseline performance was found to be associated with positive outcomes in both *correct-steps* and *correct-patients* studies. Intervention setting and intervention provider showed a relative strong dependency (Fisher exact test: 0.00296). This might explain why inclusion of one of the two factors may have led to exclusion of the other. The higher number of technique steps evaluated being positively correlated to the effectiveness of the intervention might point to a methodological issue. A potential explanation for this correlation could be that interventions are improving parts of the patient’s technique which are not measured by all studies. Considering the large variability of outcome measures in use, ranging from five to eleven different steps in inhaler technique,^24, 45, 48^ this seems plausible. Equally important are the factors which were found to not be associated with improvements in inhaler technique. Duration and type of intervention, individual or group based, did not have a significant impact on outcomes. This bears important consequences for health-economic decisions in clinical practice, as less time-consuming and group interventions can be selected without sacrificing effectiveness. The addition of general disease education, or in more general terms, embedding the intervention in a multi-component intervention did not provide a benefit for improving inhaler technique either. Contrary to reports of multi-component interventions on adherence which showed mixed results,^58^ inhaler technique did not suffer negative effects. Intervention effectiveness was shown to be independent of disease, but note that COPD-specific studies were scarce and patient populations were heterogeneous, warranting further investigation of specific subgroups (such as children and patients with low literacy). Patients benefited from interventions, irrespective of the type of inhaler they used. This is in line with previous studies, which report improvements made with multiple types of inhalers.^59^ It also confirms a previous recommendation to educate patients on their inhaler instead of switching inhalers.^60^

### Strength and limitations

To our knowledge, this is the first systematic review on educational inhaler technique interventions in asthma and COPD patients and it provides definitive evidence on their effectiveness and success factors. Especially given the current wealth of new inhalers available and insurer-driven inhaler switches, we feel this study is very relevant and timely. This systematic review was however limited to RCTs and did therefore exclude useful observational studies. A limiting factor was the wide variety of interventions and outcome measures, hampering the performance of a meta-analysis. From a clinical perspective it is unrealistic to combine the outcome effects of studies that focus on different patient populations (e.g. school children vs. adults) and implement interventions with vastly different characteristics (e.g. short video vs. interactive disease management sessions). Performing a meta-analysis would imply comparability of interventions and may lead to false interpretation of outcomes.

Assessment of relevant independent variables in the regression analysis was based on forward selection and backward elimination procedures. These procedures are sometimes referred to as data dredging methods and come with their own flaws, such as an overestimation of the variance explained by the model.^61^ Furthermore, the backward elimination models suffered from problems with multicollinearity resulting in the exclusion of several potential predicting variables. Nonetheless, considering the explorative nature of this study and the lack of clinical guidance on relevant independent predictors of improvement of inhaler technique over baseline, this methodology was assessed to be a relevant option to use.

The vast majority of studies showed a positive effect of their intervention on inhaler technique, a warning marker for potential publication (or reporting) bias. An alternative explanation for the positive results could be the relatively short follow-up time of most studies. Lastly, it should be noted that studies were conducted by well-trained healthcare professionals with plenty of time available. In clinical practice however, available consultation time, knowledge and skills regarding inhalers among healthcare professionals are often limited, highlighting that well-trained intervention deliverers with sufficient time available are essential.^62, 63^

### Implications for future research, policy and practice

Focusing efforts and resources on educational interventions could result in improved inhaler technique and clinical outcomes in asthma and COPD patients.^64^ This is an important finding underlining the value of educational interventions. Switching of inhaler devices is associated with several disadvantages to the patient, such as an increase in the number of errors made and reduced compliance.^65^ In this light, clinicians may prefer to opt for an educational intervention to improve inhaler technique of the device currently in use by the patient.

The effectiveness of interventions holds true for patients with an insufficient initial technique, whereas interventions may be less valuable for patients with an already moderate to good technique. Therefore, the patient population targeted by an intervention could affect its cost-effectiveness. Unfortunately, only few cost-effectiveness studies have been conducted on improving inhaler technique in COPD ^66^ and asthma.^67, 68^ Considering constraints on budgets and time available, clinicians may wish to provide intervention on inhaler technique to patients who have been identified to suffer from a poor inhaler technique, instead of indiscriminately providing these interventions to a more general patient population. Regular reviewing of inhaler technique is a recommendation that has been voiced previously ^65^ and enables a more appropriate application of interventions.

Evidence on effectiveness of educational inhaler technique interventions in COPD patients is scarce and the rate of inhaler errors has not decreased over time.^14^ To ease comparability, we recommend that studies use a uniform method to assess inhaler technique. Unfortunately, no golden standard exists yet, but technological developments, including acoustic sound based technology and eHealth applications are promising.^69, 70^

Lastly, the positive effect of interventions seems to wane over time, stressing the need for continuous monitoring and periodically reinforcement of inhalation instructions. In conjunction with continuous monitoring and periodical retraining it may be important to match the inhaler device to the patient, ensuring a high baseline performance of inhaler technique.^71^ This could potentially reduce the need for retraining of patients.

Considering the important role of inhaler medication in asthma and COPD, future research should try to understand the type of educational interventions that could be effective in different patient groups, the optimal duration of the interventions, their maintenance and ways to improve their cost-effectiveness.

## Conclusions

Educational interventions on inhaler technique in asthma and COPD patients are effective on the short-term. Key predictors for success are patient’s initial technique and time elapsed since intervention. Disease and inhaler do not play a significant role. Periodical intervention reinforcement and longer follow-up studies, including clinical relevant endpoints and cost-effectiveness, are recommended.

## Methods

### Study design

The study design was a systematic review, performed as per PRISMA-guideline.^72^

### Inclusion and exclusion criteria

All articles reporting randomised controlled trials (RCTs) on interventions aimed at improving inhaler technique in asthma or COPD patients (no age restriction) vs. usual care, published before 31 March 2015, were eligible for inclusion. Exclusion criteria were non-English manuscripts, no asthma or COPD, non-original research, qualitative studies, non-RCT design and interventions not aimed at patients. In addition, articles that did not operationalise their outcome measures or interventions without individual components description were excluded.

### Search strategy

Manuscripts were retrieved from the Medline, Embase and CINAHL-databases. It is advisable to use a combination of both Medline and Embase as they return only partly overlapping results.^73, 74^ CINAHL was added as it provides additional coverage on the nursing subfield.

Keywords in the search strategy (please refer to e-Appendix 1 for a full overview) related to both intervention and disease. Intervention keywords included a combination of variations on ‘inhaler’ and ‘technique’ or ‘instructions’, whereas disease keywords included variations on ‘asthma’ and ‘COPD’. Disease specific keywords were based on previous publications.^58^ A high sensitivity therapy filter based on the work of the Hedges Project was selected to limit search results to clinical trials, while reducing the probability of excluding relevant studies.^75, 76^ The filter was extensively validated for all three databases included within this review and was shown to have a sensitivity of 94.6% to 99.4%.^77–79^

Initial screening based on title and abstract was conducted by one reviewer (S.K.). Afterwards, each potentially eligible full-text manuscript was independently reviewed by at least two reviewers (S.K., J.B., and M.H.). Disagreements were resolved in consensus round(s).

### Quality assessment

All included articles were independently assessed by two different reviewers (S.K., J.B., M.H., M.R.) using the Cochrane Collaboration’s tool for assessing risk of bias in randomised trials.^80^ Scoring was carried out as described in the tool’s guidelines,^74^ even though risk of performance bias was not fully applicable, due to lack of feasible blinding options of participants. This is characteristic of educational interventions. Inter-reviewer discrepancies in scoring were resolved in a consensus procedure.

### Data extraction

Study characteristics, study population, and outcomes were systematically recorded for all included articles using a pre-structured spreadsheet.

#### Study characteristics

If multiple intervention groups were included within a single study, or outcomes were separately reported for pMDI and DPI users, each group was recorded separately. Data were extracted by a single researcher (S.K.) in order to maintain consistency throughout coding. Extracted data of 10% of the included studies was validated by a second reviewer (M.H. or J.B.), based on a random sample. Extracted study characteristics included country, intervention, comparator, setting, executor, delivery form, sample size and follow-up time. Setting was categorised as community pharmacies, hospitals, schools, outpatient clinics or other. Executors were labelled as researchers, pharmacists, nurses (including assistants and community healthcare workers) or other. Delivery form was either group education or individual education. Follow-up time was recorded as the time elapsed between baseline and last measurement.

#### Study population

Study population data covered disease, inhaler, mean age and sex. Disease was either asthma or COPD. In case both asthma and COPD patients were included and outcomes were not segregated by disease, disease type was recorded as asthma and COPD. Inhaler type was generalised into two main categories, i.e. pMDI and DPI. pMDI included inhalers with and without spacers. DPI included inhalers such as Turbuhaler and Diskus. Age was originally recorded as mean age and afterwards dichotomised into children (<18 years) or adults. Sex was recorded as the percentage of males.

#### Outcomes

Outcomes were categorised a priori into two main classes: studies reporting the number of correct technique steps, and studies reporting the percentage of patients with a correct technique. For ease of reading, the former group of studies will be referred to as ‘*correct-steps*’ studies, the latter as ‘*correct-patients*’ studies. Based on the clinical experience of two of the authors (M.R.R. and T.v.d.M.) as well as opinions from an external clinical expert showed that patient handling of inhalers was in clinical practice usually evaluated on different steps. Hence, if a study reported both outcomes, preference in the data extraction was given to the number of correct steps. Furthermore, this outcome measure provided richer data. If studies also reported relevant clinical endpoints, these data were extracted as well and assessed in a descriptive manner.

### Analysis

Agreement between reviewers on inclusion eligibility was tested with Cohen’s Kappa and interpreted according to guidelines.^81^ Inter-reviewer agreement on the quality appraisal was tested with a weighted Cohen’s Kappa test which gives more weight to larger differences in ordinal values.^82^ Data were initially evaluated in a descriptive way. In a second step, linear regression models were created considering inhalation technique improvement as dependent variable. Potentially associated independent variables were tested for significant predictive value. These included year of publication, country, setting, executor, delivery form, sample size, follow-up time, disease, inhaler, age, sex and baseline performance. The number of steps evaluated, and performance required from a participant to be coded as showing correct technique were tested when applicable. Due to the difference in outcome measurement, one regression model was created for *correct-steps* studies, and another model was created for *correct-patients* studies. Both forward selection and backward elimination methods were used to assess the inclusion of independent variables, at a significance threshold of 0.05. All quantitative analyses were conducted in SPSS^®^ Statistics version 22.

## Electronic supplementary material

eAppendix 1

eAppendix 2

eAppendix 3

eAppendix 4
