# Supplementary material for: Effectiveness and success factors of educational inhaler technique interventions in asthma & COPD patients: a systematic review
Source: NPJ Prim Care Respir Med. 2017 Apr 13;27:24. doi: 10.1038/s41533-017-0022-1 (PMC5435089; doi:10.1038/s41533-017-0022-1)
Supplement: Supplementary file 1 — eAppendix 1 [file 41533_2017_22_MOESM1_ESM.docx]

# eAppendix 1: Search strategy

Therapy -
high sensitivity

Asthma [Mesh]

Asthma

COPD [Mesh]

OR

OR

OR

COPD

AND

AND

**Intervention**

Competence

Handling

Instructions

Technique

OR

OR

OR

Inhalation

Inhaler

OR

AND

**Disease**

**Study type**

*eFigure 1 – High level overview of the search strategy*

**MEDLINE (via PubMed)**

*Keywords*

((inhalation[Title/Abstract] OR inhaler[Title/Abstract]) AND (competence[Title/Abstract] OR handling[Title/Abstract] OR instructions[Title/Abstract] OR technique[Title/Abstract])) AND ("Asthma"[Mesh] OR asthma[Title/Abstract] OR "Pulmonary Disease, Chronic Obstructive"[Mesh] OR COPD[Title/Abstract] OR "Emphysema"[Mesh] OR "Pulmonary Emphysema"[Mesh] OR "Bronchitis, Chronic"[Mesh] OR "Lung Diseases, Obstructive"[Mesh]) AND ((clinical[Title/Abstract] AND trial[Title/Abstract]) OR clinical trials as topic[MeSH Terms] OR clinical trial[Publication Type] OR random*[Title/Abstract] OR random allocation[MeSH Terms] OR therapeutic use[MeSH Subheading])

*Filters*

Language: English

Publication dates: to 2015/03/31

**CINAHL (via EBSCO)**

*Keywords*

((inhalation OR inhaler) AND (competence OR handling OR instructions OR technique)) AND (MM("Asthma" OR "Pulmonary Disease, Chronic Obstructive" OR "Emphysema" OR "Bronchitis, Chronic" OR "Lung Diseases, Obstructive") OR ("Asthma" OR "COPD"))

*Filters*

Clinical Queries: Therapy – High Sensitivity

English Language

Published Date: -20150331

**EMBASE (via Ovid)**

*Keywords*

(('inhalation'.ti,ab OR 'inhaler'.ti,ab) AND ('competence'.ti,ab OR 'handling'.ti,ab OR 'instructions'.ti,ab OR 'technique'.ti,ab)) AND (('asthma'/exp OR 'chronic obstructive lung disease'/exp OR 'lung emphysema'/exp) OR ('Asthma'.ti,ab OR 'COPD'.ti,ab))

*Filters*

Clinical Queries: Therapy (maximizes sensitivity)

English Language

Date of Publication: - 31 Mar 2015
